# Periventricular Nodular Heterotopias Induced-Seizures in an Adolescent

**DOI:** 10.7759/cureus.75272

**Published:** 2024-12-07

**Authors:** Andreia Fernandes, Mafalda J Pereira, Íris Oliveira, André M Travessa, José Drago, Carla Mendonça

**Affiliations:** 1 Pediatrics, Unidade Local de Saúde do Algarve-Hospital de Faro, Faro, PRT; 2 Genetics, Unidade Local de Saúde Santa Maria e Universidade de Lisboa, Faculdade de Medicina, Lisboa, PRT; 3 Radiology, Unidade Local de Saúde do Algarve-Hospital de Faro, Faro, PRT

**Keywords:** clinical heterogeneity, flna-variant, genetic testing, periventricular nodular heterotopias, seizures

## Abstract

A female adolescent with no relevant past history was admitted to the Pediatric Emergency Department with two episodes of seizures without trauma, fever, or other symptoms. Head-MRI revealed bilateral subependymal nodular irregularities lining the lateral ventricles, with similar signal evolution to grey matter, confirming the diagnosis of periventricular nodular heterotopias (PVNH). Genetic testing revealed a Filamin A (*FLNA)* variant; family studies were negative. Further investigation excluded associated comorbidities.

PVNH is a rare condition caused by an abnormality in neuronal migration and is part of a phenotypically and genetically heterogeneous group of disorders. *FLNA* variants are PVNH’s most common cause and are responsible for classical neuroradiologic imaging, characterized by bilateral symmetrical nodules with predominantly anterior and mid-body distribution along the linings of lateral ventricles. Classical PVNH typically affects females presenting with seizures in mid-adolescence, with normal/mildly impaired cognition; males are more severely affected. *FLNA*-induced PVNH may be associated with comorbidities, which may be severe. Other rarer genetic causes combine PVNH with complex brain or systemic abnormalities, resulting in developmental delay and a worse prognosis.

Genetic diagnosis is essential for adequate counseling, follow-up, seizure control, vigilance, and treatment of comorbidities, as they may have a great impact on the patient’s quality of life.

## Introduction

Periventricular nodular heterotopias (PVNH) is a rare condition caused by an abnormality in neuronal migration during fetal brain development, resulting in the formation of grey matter nodules along the linings of the lateral ventricles [1-2]. These nodules can be morphologically different, ranging in size and distribution, and may be unilateral, bilateral, focal, or diffuse [2-4]. Head magnetic resonance imaging (MRI) is the most sensitive neuroimaging method for diagnosis because a computerized tomography (CT) scan might not show any abnormalities [3,5]. Previously, PVNH was categorized into two types: isolated PVNH, where patients experienced seizures but exhibited normal cognition and development, and PVNH associated with complex brain or systemic abnormalities, which was linked to developmental delays, severe intellectual disabilities, and early mortality. [3,6]. This clinical variability in patients with identified PVNH on neuroimaging was previously not fully understood, but recent advances in Genetics have shown that PVNH is part of a phenotypically and genetically heterogeneous group of disorders resulting from de novo or familial mutations [4,7,8].

Here, the authors report a case of a rare cause of seizures in pediatrics, specifically in adolescence, aiming to bring attention to this condition, its clinical features, neuroimaging findings, genetic testing, and associated comorbidities.

## Case presentation

**Figure 1:**
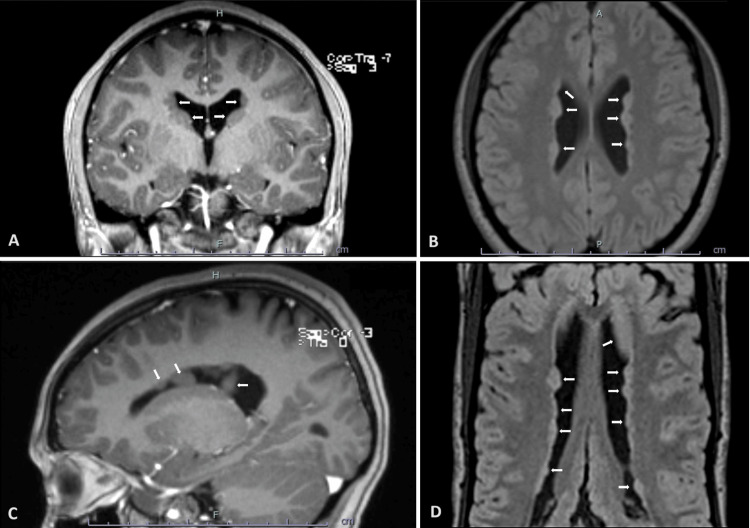
Patient’s head magnetic resonance imaging. Images of our patient’s head magnetic resonance imaging, on coronal (A), axial (B and D) and sagittal (C) planes, showing bilateral subependymal nodular irregularities lining the lateral ventricles. These nodules exhibit a similar signal evolution to that of the grey matter, confirming the diagnosis of periventricular nodular heterotopias.

A 17-year-old female adolescent with no relevant history was admitted to the Pediatric Emergency Department with two brief episodes of loss of consciousness, upward eye deviation, and tonic-clonic movements of the limbs, followed by postictal confusion. There was no history of previous or current infection, associated trauma, headache, or other symptoms. She also reported a brief episode of left-sided facial and tongue paresthesia in the previous month. Besides slurred speech, physical and neurological examinations were normal. Laboratory tests were unremarkable (complete blood count, blood gases analysis, lactate, blood alcohol content, and urine toxicology screening), and the electroencephalogram was normal. Head CT scan showed irregularity of the lining of the lateral ventricles, with no calcifications, and MRI identified multiple bilateral subependymal nodular irregularities lining the lateral ventricles, with similar signal evolution to grey matter (Figure 1), confirming the diagnosis of subependymal heterotopias or PVNH. Whole-exome sequencing revealed a *FLNA* variant (NM_001110556.1), c.7896G>A p.(Trp2632*), classified as likely pathogenic, and family studies were negative, confirming a ‘de novo’ mutation. Further investigation excluded associated comorbidities, namely cardiovascular disease. Initial seizure control was challenging, requiring frequent antiepileptic drug adjustments, but currently, at follow-up, epilepsy is well controlled with levetiracetam, perampanel, and valproate.

## Discussion

Pathogenic variants in the Filamin A (*FLNA*) X-linked gene, which encodes the cytoskeletal protein filamin A, which is important for neuronal migration, vascular function, and connective tissue integrity [7], appear to be the most common cause of PVNH [4]. *FLNA* variants account for the great majority of familial PVNH but explain only 26% of the sporadic cases [2]. Mutations in this gene, mostly loss-of-function variants and missense variants affecting the N-terminal actin-binding domain, are the most frequent cause of the so-called classical PVNH [2]. Classical PVNH, the commonest neuroimaging presentation of PVNH, is characterized by bilateral symmetrical nodules with predominantly anterior and mid-body distribution along the linings of the lateral ventricles, with limited extension to the occipital horns [3-4,9]. Typically, patients with classical PVNH are females, presenting with recurrent seizures (frequently focal, but that may generalize), most commonly beginning in mid-adolescence [2-3,9]. These seizures may be refractory to treatment [2-3,9], sometimes requiring antiepileptic polytherapy, as in our patient's case. Surgery may also be indicated in eligible cases [5]. In addition, physical examination and cognition are usually normal, but there may be mild intellectual disability, mostly involving difficulties in reading or spelling, processing speed, and executive functions [1-4,9]. Males are more severely affected than females, with a high intrauterine and perinatal mortality. Males with classical PVNH may survive due to the presence of variants allowing some expression/function of *FLNA* or by the occurrence of mosaicism [2,4,7,8]. Phenotypic expression is variable in both females and males, and this can be partially explained by the pattern of X inactivation in females (with some females being asymptomatic) and by the severity and type of variant in both sexes [2,9]. However, some phenotypic variability is not yet understood.

In our patient's case, the bilateral nodules with anterior and mid-body distribution in the linings of the ventricles on neuroimaging and the fact that the patient is a female, presenting with focal and generalized seizures, with normal physical examination and cognition raised the hypothesis of *FLNA*-associated classical PVNH, which was posteriorly confirmed by genetic testing.

*FLNA*-associated PVNH may also present with an Ehlers-Danlos-like phenotype with cardiovascular and connective tissue abnormalities, such as cardiac valve disease and vascular defects or dilation (mainly aortic), and small and large joint hypermotility [2-3,9]. The expression of this Ehlers-Danlos-like phenotype is inconstant, even in patients with the same variant, but the cause for this is still not fully understood [10]. Other *FLNA *variants are associated with other extracerebral manifestations besides PVNH, like chronic obstipation, chronic obstructive lung disease, and platelet abnormalities [8,9]. On the other hand, the oto-palato-digital (OPD) syndrome spectrum results from gain-of-function *FLNA* variants and is usually not associated with PVNH; exceptionally PVNH and OPD syndrome spectra may coexist when both loss-of-function and gain-of-function variants are present [2,9].

Rarer genetic causes of PVNH include loss-of-function pathogenic variants in the ARFGEF2 gene and pathogenic variants in the FAT4, DCHS1, NEDD4L, MAP1B, and AKT3 genes [2]. In these cases, PVNH can often be distinguished from classical PVNH because of the inheritance pattern, the different distribution of nodules within the ventricles on neuroimaging, and the presence of other neuroradiological features (microcephaly, polymicrogyria, hippocampal malformation, cerebellar hypoplasia) and extracerebral syndromic characteristics (limb abnormalities, dysmorphic facial features, etc.) [2,4,9]. PVNH associated with variants in these genes usually results in early-onset seizures, severe developmental delay, and intellectual disability, with poor outcomes [9]. Some patients with PVNH have no identifiable genetic etiology.

Apart from PVNH, our patient had no other apparent comorbidities. Nevertheless, she will continue to be monitored for emerging symptoms or signs due to the possibility of comorbidities appearing throughout her life, particularly cardiovascular malformations [9,10]. Follow-up in patients with classical-PVNH is not well-established in the literature. However, the authors recommend monitoring seizure control under antiepileptic therapy, the occurrence of new symptoms or signs, and serial echocardiograms even if the patients remain asymptomatic. Additionally, genetic counseling and family planning should be provided.

## Conclusions

With this case report, the author's goal is to raise attention to PVNH, an unusual cause of seizures, summarizing its clinical features and thus facilitating its recognition and clinical approach. Although PVNH diagnosis is based on clinical manifestations and neuroimaging findings, it is a complex clinical entity that should be confirmed by appropriate genetic testing in all patients. This is important not only for genetic counseling and family planning but also for the identification of possible associated comorbidities, which may be asymptomatic but have an impact on prognosis, adequate follow-up, prevention of complications, and treatment, ultimately improving patients' quality of life.
